# Redefining Obesity: A Narrative Review of Diagnostic Evolution, Therapeutic Strategies and Psychosocial Determinants

**DOI:** 10.3390/healthcare13161967

**Published:** 2025-08-11

**Authors:** Artur Przybyłowski, Michał Górski, Weronika Gwioździk, Renata Polaniak

**Affiliations:** 1 Department of Human Nutrition, Faculty of Public Health in Bytom, Medical University of Silesia in Katowice, 40-055 Katowice, Poland; 2 Department of Chronic Diseases and Civilization-Related Hazards, Faculty of Public Health in Bytom, Medical University of Silesia in Katowice, 40-055 Katowice, Poland; 3 Department of Epidemiology, Faculty of Health Sciences in Bytom, Medical University of Silesia in Katowice, 41-902 Bytom, Poland

**Keywords:** obesity, chronic disease, body weight stigma, GLP-1 receptor agonists, multidisciplinary care, health equity, bariatric surgery, psychosocial determinants

## Abstract

Obesity is increasingly recognized as a chronic, relapsing, and multifactorial disease requiring individualized care. This narrative review synthesizes recent developments in the definition, diagnosis, and treatment of obesity, highlighting key shifts in diagnostic frameworks (e.g., ICD-11), advances in pharmacotherapy, and the psychosocial dimensions shaping care engagement. Limitations of BMI, the impact of stigma, and structural inequities are discussed as barriers to effective management. By integrating clinical, psychological, and societal perspectives, this review advocates for a multidimensional, stigma-informed, and equity-oriented approach to obesity care.

## 1. Introduction

Obesity is a major global health challenge in the 21st century, with steadily rising prevalence across all age groups and regions. This condition exerts a profound impact not only on individual health outcomes but also on healthcare systems and economic productivity. Once regarded primarily as a consequence of poor lifestyle choices, obesity is now recognized as a complex, chronic disease with multifactorial origins, encompassing physiological, metabolic, psychological, and environmental components [1,2].

A key development in this reconceptualization was the formal classification of obesity as a chronic disease under ICD-11, which emphasized its long-term character and systemic implications [3]. This shift in definition has prompted a reassessment of diagnostic frameworks, treatment standards, and public health strategies, challenging the adequacy of traditional indicators such as body mass index (BMI), waist circumference, and waist-to-hip ratio as standalone measures.

Despite its growing recognition as a chronic condition, there is ongoing debate about how obesity should be diagnosed, managed, and contextualized within broader health and social policy frameworks. The complexity of obesity extends beyond clinical parameters and includes substantial sociocultural and psychological dimensions, such as internalized stigma, inequities in access to care, and the influence of socioeconomic status on treatment outcomes [4,5].

The global prevalence of obesity has risen dramatically, becoming a major contributor to both morbidity and mortality worldwide. According to the World Health Organization (2022), 59% of adults in the European Region are overweight or obese, with nearly 23% classified as obese. Rates of obesity exceed 70% among men in some Mediterranean and Eastern European countries, with the highest prevalence observed in populations with lower socioeconomic status [6]. This reflects a growing global epidemic, particularly in low-income nations, where obesity continues to escalate.

An analysis conducted across 12 European countries revealed that nearly half of adults (48.1%) were overweight or obese, with Greece and Romania showing the highest obesity rates. Notably, there has been a rising trend in Eastern and Northern European countries since 2010, while countries like Italy and France reported a decline [7].

On a global scale, while high-income countries may have experienced a plateau in obesity rates, the epidemic continues to escalate in low-income populations [8]. In South Africa, obesity rates are particularly alarming, with 31% of men and 68% of women classified as obese. This trend highlights the growing health burden in middle-income countries, especially among urban populations [9].

In South Asia, a meta-analysis of 152 studies found that 6.6% of adults are obese, with the highest rates in Bangladesh, where 8.9% of the population is affected. The prevalence of obesity is particularly concerning, as these countries face rapid urbanization and dietary changes [10]. Additionally, across South and Southeast Asia, obesity is on the rise, with countries like India already seeing adult obesity rates as high as 40.3% [11].

These findings underscore the urgent need for region-specific public health strategies to address the obesity epidemic, considering the socio-economic, cultural, and healthcare system differences across the world.

This narrative review aims to synthesize and critically examine recent literature on the redefinition and diagnosis of obesity, with particular attention to its implications for clinical practice, policy, and social perception. The central research question guiding this review is: How does the reclassification of obesity as a chronic disease influence its clinical interpretation, diagnosis, and management? Particular emphasis is placed on the following themes: revisions and limitations of current diagnostic criteria [3,12]; the role of body weight stigma, internalized bias, and sociocultural determinants [13,14]; advances in pharmacological and surgical interventions [15,16]; shifts in international definitional frameworks (e.g., ICD-11, DSM-5); and the controversy surrounding the obesity paradox and implications for disease classification [17,18]. This narrative review was conducted to provide an integrative and critical analysis of recent advances in the definition and diagnosis of obesity, with particular attention to its clinical, psychosocial, and sociopolitical dimensions. Given the interdisciplinary and evolving nature of obesity research, a narrative approach was deemed the most suitable methodology to capture both conceptual developments and the complexity of current debates [18].

## 2. Materials and Methods

### 2.1. Literature Selection Strategy

This narrative review was based on a relevance-oriented and selective search strategy conducted in the PubMed, Scopus, and Google Scholar databases. The search covered the period from January 2020 to June 2025 and focused on English-language literature. Search terms included combinations such as “obesity diagnosis”, “GLP-1 agonists”, “obesity stigma”, “bariatric surgery outcomes” and “ICD-11 and obesity”.

A total of 94 sources were included, comprising peer-reviewed journal articles, systematic and narrative reviews, expert consensus statements, clinical guidelines, doctoral and master’s theses, and institutional publications from recognized health authorities (e.g., WHO, EASO). Literature was selected based on its relevance to the evolving conceptualization of obesity and its diagnostic, therapeutic and psychosocial implications. Particular emphasis was placed on identifying sources addressing updated diagnostic criteria, sociocultural determinants such as stigma, emerging pharmacological and surgical treatments, definitional transitions in guidelines (e.g., ICD-11, DSM-5) and debates around the obesity paradox and disease status.

Although no systematic protocol was applied, the search strategy emphasized methodological rigor and clinical relevance. Gray literature (e.g., WHO and EASO publications) was included when pertinent to medical or policy-oriented discussions.

### 2.2. Inclusion Criteria

Studies were included if they met the following criteria:

- Published between January 2020 and June 2025;
- In English;
- Addressed clinical, psychological, public health, or policy-related aspects of overweight and obesity;
- Presented original research, systematic or narrative reviews, guidelines, consensus statements, or academically credible gray literature.

### 2.3. Exclusion Criteria

Studies were excluded if they met the following criteria:

- Focused on unrelated conditions (e.g., underweight, cachexia);
- Lacked peer review or sufficient methodological detail (e.g., unclear study design, absence of data sources, or no methodological description);
- Were duplicative and failed to offer novel conceptual or empirical insight.

Although formal risk-of-bias tools such as GRADE or AMSTAR were not applied—given the narrative character of this review—methodological transparency and peer-reviewed status were important selection factors. Exceptions were made for institutional reports and academic theses when their relevance to clinical or policy-related discourse was clearly established.

### 2.4. Analytical Framework

The selected literature was analyzed thematically and grouped into the following domains:

- Redefining Obesity: Diagnostic Models and Classification Debates;
- Clinical and Functional Perspectives on Risk and Assessment;
- Psychosocial Burden, Stigma, and Structural Inequities;
- Innovations in Therapy: Pharmacology, Multidisciplinary Care, and Policy Implications.

These domains were not pre-specified but emerged inductively during the synthesis of sources. The analysis aimed not only to summarize findings but also to critically address conceptual tensions, knowledge gaps, and methodological limitations in the literature. By integrating diverse types of evidence and disciplinary lenses, this review offers a contextualized and multifaceted understanding of obesity as a chronic and complex condition.

## 3. Discussion

### 3.1. Evolution of Clinical Obesity

The clinical understanding of obesity has undergone a substantial conceptual transformation in recent years. As diagnostic frameworks and theoretical models continue to evolve, obesity is recognized not merely as a result of lifestyle choices but as a heterogeneous, chronic disease with complex biological and psychosocial underpinnings. This growing recognition has prompted the need for more precise, individualized definitions that account for both physiological dysfunction and broader determinants of health. Within this review, the evolution of diagnostic standards provides the foundation for examining shifts in how clinical obesity is defined and managed in contemporary healthcare.

#### 3.1.1. Current Definitions and Limitations of BMI

Emerging definitions of obesity increasingly move beyond the simplistic view of excess body weight or fat accumulation. A widely cited proposal defines obesity as a disease characterized by abnormal or excessive adipose tissue that impairs health and physiological function [3]. This perspective emphasizes the biological, metabolic, and endocrine disruptions associated with adiposity, rather than focusing solely on quantitative thresholds like BMI. The 2022 Korean clinical practice guidelines stress using comorbidities as the basis for diagnosing obesity and abdominal obesity, shifting the focus away from purely anthropometric thresholds [19].

While widely discussed, this definition has elicited criticism. In its formal response, the European Association for the Study of Obesity (EASO) acknowledged the value of updating definitional frameworks but voiced concern over the operational limitations of Rubino et al.’s [3] proposal—specifically, the lack of measurable diagnostic thresholds and the potential for inconsistent clinical application [20]. This debate exemplifies the ongoing struggle to balance conceptual accuracy with clinical utility.

Body Mass Index (BMI) has long been used as a standard metric for diagnosing obesity; however, it offers only a crude estimation of health risk and fails to differentiate between adipose tissue and lean body mass. For instance, individuals with high muscle mass may be misclassified as obese, while those with low muscle mass and high adiposity may be inaccurately categorized as within a healthy range. This lack of specificity undermines BMI’s diagnostic validity at the individual level [3,12].

To address these shortcomings, contemporary guidelines increasingly recommend complementing BMI with additional anthropometric and body composition measures, such as waist circumference, waist-to-hip ratio, and body fat percentage. Furthermore, standardized BMI cut-off points may yield misleading prevalence estimates due to interethnic variations in body fat distribution and age-related physiological changes such as sarcopenia. For example, the World Health Organization (WHO) defines obesity using BMI thresholds of 30 kg/m^2^, while the Asia-Pacific region employs lower cut-offs (≥27.5 kg/m^2^) to account for differences in body fat distribution [20]. This underscores the importance of considering regional and ethnic factors in obesity diagnosis. These indicators improve risk stratification—particularly among individuals with normative BMI values who may nonetheless present with metabolically unhealthy profiles [12,21].

Furthermore, standardized BMI cut-off points may yield misleading prevalence estimates due to interethnic variations in body fat distribution and age-related physiological changes such as sarcopenia. For older adults, this can result in underdiagnosis of obesity-related risks, as BMI does not adequately capture fat-free mass loss or increased visceral adiposity [22,23,24]. Direct adiposity assessments, when combined with anthropometric markers, offer a more accurate and individualized approach to diagnosis and treatment planning [25]. Moreover, various anthropometric tools are used to assess obesity beyond BMI. Waist circumference (WC) and waist-to-hip ratio (WHR) are key indicators, with WC being particularly useful in identifying central or visceral obesity, a major risk factor for metabolic diseases [23,26]. Additionally, skinfold calipers are often employed to estimate subcutaneous fat, providing an indirect measure of body fat percentage [27]. These methods, while more complex, allow for more precise health risk assessments than BMI alone, especially in populations where BMI may not fully capture the extent of obesity-related health issues, such as older adults or individuals from Asian populations [28].

These refinements are reflected in the ICD-11 definition of obesity as a chronic disease in which excess adiposity increases the risk of associated comorbidities. This redefinition marks a conceptual shift away from viewing obesity as a behavioral issue and toward recognizing it as a pathophysiological condition that warrants sustained clinical attention [3,20].

Recognizing obesity as a chronic disease aligns its management with that of other long-term conditions, promoting early intervention, continuity of care, and multidisciplinary coordination. Such a framework encourages policy-level changes to support access to pharmacological and surgical treatments, as well as the integration of obesity management into broader chronic disease prevention programs [20].

Importantly, adverse health effects associated with obesity often occur before conventional BMI thresholds are reached, suggesting that reliance on BMI alone may delay diagnosis and intervention. Direct measures of adiposity—particularly waist circumference and body fat percentage—can more effectively identify at-risk individuals, enabling earlier, targeted clinical responses [3,17]. This issue is further complicated by the existence of metabolically healthy obesity (MHO), a phenotype where individuals with elevated BMI may not exhibit expected metabolic dysfunctions, yet still carry long-term health risks. Recent meta-analyses confirm that MHO is not benign and often progresses to metabolically unhealthy states over time [29].

These limitations have contributed to the emergence of the so-called obesity paradox, wherein individuals with higher BMI scores paradoxically demonstrate better clinical outcomes in certain chronic disease contexts [17]. This paradox was substantiated in a large-scale cohort study, which found that overweight and obese patients hospitalized with acute infectious diseases exhibited significantly lower short- and long-term mortality compared to those with normal or low BMI [30]. This phenomenon may be explained by confounding factors such as reverse causation, selection bias, and the presence of sarcopenic obesity. Moreover, it underscores the importance of assessing adiposity phenotypes rather than relying solely on BMI classifications [18].

Phenotypic diagnostic models prioritize observable health impairments, metabolic risk, and organ-specific dysfunction over simplistic body weight thresholds. They allow clinicians to tailor intervention strategies based on individual risk profiles and functional markers, thereby enhancing the clinical utility of diagnostic practices in obesity care. These limitations underscore the importance of incorporating additional, direct measures of adiposity and metabolic health.

Table 1 summarizes the definitions proposed by key health authorities and expert panels, highlighting their conceptual differences and practical implications. Section 3.1.3 explores the diagnostic tools and criteria currently recommended to address these gaps and to support early, individualized risk assessment.

Given the increasing recognition of obesity’s complexity, there is a growing consensus that existing definitions—and the diagnostic tools derived from them—must evolve to better reflect the multifactorial nature of the condition. As such, refining diagnostic frameworks is critical to improving both clinical accuracy and treatment personalization. Moving beyond BMI-centric perspectives is a crucial step in this process, and the next section elaborates on the limitations of BMI as a diagnostic tool, highlighting the need for a more nuanced, phenotype-based approach to clinical classification.

#### 3.1.2. Diagnostic Tools and Criteria

Recent advances in obesity diagnostics have prompted a re-evaluation of traditional classification standards, particularly the long-standing reliance on BMI. While BMI offers a population-level proxy for body weight status, it fails to differentiate between fat mass and lean body mass. These limitations are particularly pronounced in older populations, where sarcopenia may mask underlying fat accumulation [3].

To overcome these diagnostic inaccuracies, current expert guidelines recommend the inclusion of direct adiposity measures alongside anthropometric indices such as waist circumference (WC) and waist-to-hip ratio (WHR). Both WC and WHR have been shown to better reflect visceral fat distribution and are more strongly associated with cardiometabolic risk than BMI alone [3,35,36]. Techniques such as bioelectrical impedance analysis (BIA) and dual-energy X-ray absorptiometry (DEXA) further enhance the clinical evaluation of obesity, particularly among individuals with normal BMI values. DEXA offers high precision in quantifying fat mass, lean mass, and bone mineral content, making it especially valuable for tracking changes during weight loss interventions [25]. In contrast, while BIA is widely accessible, it tends to underestimate fat percentage in overweight individuals and shows limited agreement with more advanced methods such as air displacement plethysmography or DEXA [37]. Nonetheless, integrating these modalities—particularly DEXA and anthropometric measures—can provide a comprehensive picture of metabolic risk and support more effective clinical decision-making [22,26].

Interest is also growing in using body fat percentage (%BF) as a diagnostic marker. One proposal [38] recommends sex-specific cut-off values of ≥25% for men and ≥35% for women to define obesity. While this approach offers greater physiological specificity, its clinical adoption is limited by the absence of standardized international guidelines.

The Obesity Medicine Association (OMA) has called for a shift away from BMI as a solitary diagnostic tool toward a more individualized, multidimensional assessment strategy. A recent clinical practice statement recommends that obesity diagnosis should integrate measures of body composition, metabolic status, and functional impact [39]. A conceptual distinction is now made between pre-clinical obesity-defined by excess adiposity without organ dysfunction clinical obesity, where physiological impairments are present [3]. This two-tiered model supports early detection and intervention, similar to frameworks used for other chronic conditions. However, diagnostic accuracy is not only a matter of anthropometric precision but also of contextual relevance. Psychological, social, and systemic factors increasingly inform diagnostic decisions and treatment planning, as outlined in the next section.

#### 3.1.3. Integrative and Interdisciplinary Diagnostic Models

Incorporating psychosocial and personality profiles into obesity diagnostics is essential. Mental health issues, such as depression, anxiety, and post-traumatic stress disorder (PTSD), significantly contribute to the development of obesity and influence treatment outcomes. Pre-bariatric psychological assessments often reveal comorbidities, particularly within the domains of detachment and negative affectivity, as classified by ICD-11 and DSM-5 [36]. Research has demonstrated that individuals with mental health conditions, such as PTSD or depression, are at greater risk of obesity and face challenges in adhering to treatment plans [32]. Moreover, older patients and those using antidepressants or antipsychotics are more likely to exhibit personality dysfunctions, further complicating weight management efforts [40].

Mental health issues and psychiatric comorbidities are particularly relevant to obesity treatment because they interact with other psychosocial factors such as body image and social support. Body weight stigma and internalized body weight bias must be addressed to ensure effective management. The consequences of weight bias include worsened psychiatric outcomes, reduced quality of life, and lower healthcare utilization, all of which increase the risk of long-term health complications. Importantly, research shows that weight stigma can affect individuals irrespective of their BMI, underscoring the need for universal action in clinical and everyday environments [41].

Obesity diagnostic systems should align with healthcare equity principles and address the social determinants of health, such as socioeconomic status, which significantly contribute to obesity risk. Consistent diagnostic criteria across disciplines can improve clarity and consistency, although the effective implementation of such standards requires resources for professional training. Strengthening primary care capacity is crucial, as primary care providers are often the first point of contact for most patients [38].

Recent studies emphasize the importance of standardized obesity care through systematic documentation of body type and obesity grade, which can improve diagnostic quality and healthcare outcomes. This approach supports accurate diagnosis and facilitates individualized treatment planning, particularly in marginalized populations [39]. Furthermore, an interdisciplinary diagnostic team—including medical, psychological, and public health professionals—is essential for evaluating obesity, as the condition’s multifactorial etiology spans endocrinology, psychology, and population health [42]. These diagnostic advances, which span physiological, psychological, and structural domains, create a foundation for more precise and individualized therapeutic strategies. The diagnostic criteria for obesity according to the organization are presented in Table 2.

Understanding how diagnostic heterogeneity interacts with treatment planning is key to optimizing outcomes. With this integrated diagnostic approach, the next crucial step is identifying the most effective treatment options for patients with obesity. In the following section, we will explore the range of treatment approaches currently available, each shaped by the evolving definition of obesity and the psychosocial context in which care is delivered.

### 3.2. Treatment Approaches

Effective obesity management requires a tailored approach that integrates lifestyle, medical, and surgical strategies. While lifestyle modification remains the foundation, its long-term efficacy depends on systemic support. The integration of updated diagnostic criteria and awareness of psychosocial dynamics enhances the relevance of each treatment modality. Accordingly, the following subsections detail specific therapeutic strategies, each shaped by the evolving understanding of obesity’s complexity. Furthermore, effective clinical interventions cannot be decoupled from the psychosocial and structural determinants that shape disease expression, healthcare access, and patient engagement. It is essential to recognize that interventions should be multifaceted, addressing not only biological factors but also the broader societal influences on health.

Lifestyle interventions are the cornerstone of obesity management. These interventions typically focus on dietary changes, increasing physical activity, and addressing eating behaviors. Dietary changes should involve a reduction in caloric intake, increased consumption of fiber, fruits, and vegetables, and a reduction in saturated fats and sugars. Physical activity should be encouraged, with a target of at least 150 min per week of moderate-intensity aerobic exercise, such as walking, swimming, or cycling. Psychological interventions, such as cognitive-behavioral therapy (CBT), play a critical role in helping patients address the psychological challenges related to obesity, including emotional eating and unhealthy eating habits. Several studies have shown that lifestyle interventions that combine diet, physical activity, and psychological support can yield significant short-term weight loss benefits and improvements in cardiovascular health and metabolic parameters [46].

Obesity treatment requires a multilevel approach that extends beyond pharmacotherapy to include educational actions, social support, psychological interventions, and public health policies. Educational initiatives on healthy lifestyle choices, alongside social support, have been shown to improve long-term weight loss outcomes. Public policies, such as improving access to healthy food, enhancing physical activity infrastructure in cities, and running public health campaigns, are essential for reducing obesity rates at the population level. Recent reviews underscore the necessity of integrating social support and environmental changes with individual behavioral interventions to combat the obesity epidemic more effectively [2,47]. Public health strategies and systemic interventions, including policy reform and societal engagement, are crucial to creating an environment conducive to healthy behaviors, ensuring that long-term weight management is not solely the responsibility of the individual.

#### 3.2.1. Lifestyle-Based Strategies and Barriers to Sustainable Weight Loss

Lifestyle interventions have historically served as the cornerstone of obesity management. While still essential, these approaches require substantial refinement. Early strategies were grounded in the “willpower” paradigm, emphasizing individual responsibility. Individuals living with obesity were expected to reduce caloric intake and increase physical activity. However, accumulating evidence reveals that biological mechanisms—including hormonal, metabolic, and neurobehavioral adaptations—predispose individuals to body weight regain following caloric restriction or increased energy expenditure [48,49]. These physiological responses, such as persistent appetite dysregulation, reduced energy expenditure, and adipose tissue “memory”, undermine long-term success and may explain the widespread recurrence of obesity after initial weight loss.

Lifestyle interventions typically involve structured dietary changes, increased physical activity, and behavioral counseling. These strategies have shown effectiveness in producing short-term improvements in weight, glycemic control, and cardiovascular risk factors. However, the long-term sustainability of weight loss remains limited. Estimates suggest that more than 80% of individuals who lose weight through diet and lifestyle intervention regain some or all of it within five years, with the most rapid regain occurring within the first 6 to 24 months [48,49]. This limitation underscores the importance of addressing the contextual and physiological drivers of weight regain.

Recent reviews highlight that interventions focused solely on individual behavior are insufficient unless accompanied by supportive environmental, social, and policy-level changes [49]. Structural barriers such as limited access to healthy foods, unsafe neighborhoods, and economic constraints can significantly impede long-term adherence. Therefore, lifestyle strategies should be integrated with broader systemic efforts that acknowledge the biological and societal complexity of obesity. This approach is essential for ensuring that patients receive not only individual guidance but also the community and policy-level support necessary for sustained weight management.

#### 3.2.2. The Role of Stigma and Mental Health

Body weight stigma represents a significant barrier to achieving long-term health success. Individuals with overweight or obesity often face discrimination and blame in both healthcare and social settings, which can lead to avoidance of these environments altogether [50]. Such experiences may contribute to maladaptive coping mechanisms, such as reduced physical activity, poor dietary management, stress-related eating, and avoidance of healthcare services [50]. Research consistently shows that body weight stigma is linked to decreased motivation and engagement, particularly in weight management programs. This relationship significantly impacts treatment adherence and outcomes.

Interventions that integrate psychosocial and behavioral counseling have shown promise in addressing these challenges. For instance, motivational interviewing, trauma-informed care, and family-based approaches have all demonstrated reductions in body weight stigma and improvements in health outcomes [51]. In a 2024 study on pediatric obesity, qualitative findings indicated that fostering positive health identities and addressing family dynamics can enhance resilience to stigma [51]. Additional behavioral strategies, such as positive reinforcement, goal setting, and behavior regulation techniques, have also proven effective in helping individuals overcome stigma-related barriers [1].

#### 3.2.3. Socioeconomic Barriers and Health Equity

Socioeconomic status—encompassing income, education, and employment—strongly influences an individual’s ability to engage in lifestyle interventions. People from disadvantaged backgrounds often face food insecurity, financial constraints, and limited access to healthcare, all of which hinder their participation in structured weight management programs [1,14]. Moreover, cultural and linguistic barriers can further reduce the effectiveness of interventions. To address these issues, it is crucial to tailor programs to meet the cultural and literacy needs of participants, ensuring that care is delivered equitably across diverse populations.

Recent research highlights that lifestyle interventions alone are insufficient to yield sustainable weight loss without broader systemic changes. For example, pandemic-related lockdowns demonstrated how external, systemic conditions—beyond individual control—can contribute to population-level weight gain [52]. Effective management of obesity at the population level requires multifaceted interventions that target individual, community, and societal factors. Nevertheless, even the most advanced therapeutic approaches can be undermined by unaddressed psychosocial barriers and structural inequalities. With this in mind, the following section explores current pharmacological strategies, which form a critical component of obesity care, particularly for individuals with limited responses to lifestyle interventions.

### 3.3. Medical Management

Pharmacotherapy for obesity management has significantly advanced with the development of GLP-1 receptor agonists (RA), such as semaglutide and liraglutide, as well as dual or multi-receptor agonists like tirzepatide. These medications have been shown to be more effective than other treatments in promoting clinically meaningful body weight loss. Approximately 37.5% of patients achieve at least a 10% reduction in body mass after two years of treatment, compared to placebo [34,53]. However, these benefits are accompanied by higher rates of adverse gastrointestinal events, such as constipation, vomiting, and diarrhea. For instance, the use of GLP-1 RAs is estimated to increase the absolute number of constipation and vomiting episodes by 118 and 110 per 1000 individuals over a two-year period [54]. As such, it is important to balance the potential benefits against these risks and implement appropriate mitigation strategies [54].

The goal of obesity treatment is not only weight loss but also improving overall health and reducing the risk of comorbidities such as type 2 diabetes, cardiovascular diseases, and hypertension [55]. Short-term goals typically focus on weight reduction by 5–10% and improving metabolic parameters (blood pressure, glucose, and cholesterol). Long-term goals include preventing the recurrence of obesity, maintaining weight stabilization, and enhancing quality of life. It is important to monitor patients after the active treatment phase to ensure that results are maintained.

Subgroup analyses have shown variability in the effectiveness of different GLP-1 RA agents. Semaglutide, for example, has been associated with a 96% probability of achieving at least a 10% body weight loss after two years, while liraglutide is associated with an 83% probability, and tirzepatide with 64%, highlighting the importance of selecting the most suitable agent based on individual tolerability [34]. In addition to GLP-1 monotherapy, dual and polyagonists such as tirzepatide and retatrutide have demonstrated superior body mass reduction outcomes, with clinical trials reporting mean body weight reductions exceeding 22% [15,33,34]. Tirzepatide, a dual agonist, activates both GLP-1 and GIP receptors, leading to enhanced appetite suppression and increased energy expenditure. Retatrutide, a polyagonist, targets GLP-1, GIP, and glucagon receptors, showing additional metabolic benefits and body weight reduction in individuals with metabolic dysfunction-associated steatotic liver disease (MASLD). Doses ranging from 1 mg to 12 mg have been tested, with higher doses yielding the most significant metabolic improvements. Its safety profile appears comparable to placebo [54], although the precise mechanisms behind its efficacy are still under investigation [15].

Obesity treatment should be tailored to the individual needs of the patient. For example, patients with concurrent metabolic diseases (e.g., diabetes, heart disease) require specialized approaches that take these comorbidities into account. Patients should also be offered a choice of treatment options (e.g., pharmacotherapy vs. bariatric surgery), which should align with their personal preferences, tolerability, and clinical needs [56].

The mechanisms by which GLP-1 RA and polyagonists induce weight reduction are based on their effects on appetite regulation and energy metabolism. GLP-1 agonists primarily reduce food intake and delay gastric emptying, which directly leads to decreased appetite. Polyagonists like tirzepatide enhance these effects by activating additional receptors such as GIP and glucagon. This dual activation results in even more comprehensive appetite suppression and increased energy expenditure, helping patients achieve better weight loss outcomes [15,51]. Consequently, these advances in pharmacotherapy reinforce the need for personalized treatment plans that take into account each patient’s specific needs and metabolic profile. Long-term safety, along with continuous monitoring, remains essential in developing and applying these treatment strategies effectively [50].

#### 3.3.1. Challenges in Long-Term Adherence to Pharmacotherapy

Despite the clinical promise of pharmacotherapy, its real-world application has not met expectations. Only 30–40% of patients remain on GLP-1 RA therapy after one year, compared to 85% retention rates in randomized controlled trials [16]. Approximately one in nine individuals switch between GLP-1 RA agents during the first year of treatment. Treatment discontinuation is often associated with adverse events, unmet expectations, financial barriers, and insurance coverage issues. Following cessation, many patients experience body weight regain, with a pooled mean increase of 2.2 kg (95% CI: 1.66–2.74), reducing long-term treatment effectiveness [16,42].

To improve long-term outcomes, it is critical to implement educational initiatives and adherence-promoting strategies. Integrating pharmacotherapy with dietary guidance, physical activity, and behavioral interventions has demonstrated greater effectiveness than monotherapy. For example, a randomized clinical trial found that a comprehensive intervention including liraglutide, diet, physical activity, and cognitive-behavioral therapy resulted in a mean body weight loss of 15.9% compared to 2.2% in the placebo group over 36 months [57].

Future research must prioritize long-term safety, cardiovascular risk profiles, and psychosocial dimensions of pharmacotherapy. Although GLP-1 RA drugs are associated with cardiovascular benefits in people with and without type 2 diabetes [57,58], recent meta-analyses also report increased resting heart rate and higher treatment discontinuation rates due to adverse effects [57]. Additionally, growing concern surrounds the psychosocial impact of GLP-1-based therapies. A large population-based cohort study demonstrated a slightly increased risk of anxiety, depression, and suicidal behavior in people treated with GLP-1 receptor agonists, particularly among younger patients with obesity but without diabetes [59]. These findings are echoed by mixed-methods analyses of social media platforms, which identified recurring reports of emotional detachment, fear of dependence, and body image disturbances during GLP-1 RA therapy [60]. Interindividual differences in treatment response—shaped by genetic, metabolic, and psychological factors—underscore the importance of individualized treatment strategies [21]. At present, phenotyping remains underdeveloped and requires further investigation to define its clinical applications. Ongoing trials are evaluating the optimal use of single, dual, and triple agonists and their applicability in routine clinical care [61].

Affordability and global accessibility remain major challenges. While newer agents hold potential to revolutionize obesity treatment, their limited availability in low- and middle-income countries restricts equitable access. The World Health Organization has proposed including GLP-1 RA drugs in the Model List of Essential Medicines to improve affordability and reach [61].

Multidisciplinary collaboration remains pivotal in optimizing medical management. As demonstrated in cancer care—where teams address complex issues such as tumor-related malnutrition [44]—similar interdisciplinary models should be applied in obesity treatment. Teams composed of endocrinologists, psychologists, dietitians, and other specialists can address the biological and psychological dimensions of obesity more comprehensively. Routine psychological assessments can help identify maladaptive behaviors and mental health challenges that may affect adherence and outcomes. A multifaceted, team-based approach enables early detection of comorbidities, supports follow-up, and improves the effectiveness of pharmacological therapies [54,62].

#### 3.3.2. Comparative Efficacy of Pharmacological Agents in Long-Term Trials

Recent clinical trials have provided robust data on the efficacy and safety of various anti-obesity medications. Below is a synthesis of the most relevant findings from long-term randomized studies.

In recent clinical practice, pharmacotherapy for obesity has evolved significantly. Semaglutide, a once-weekly GLP-1 receptor agonist, has demonstrated outstanding efficacy: in the STEP 1 trial, adults without diabetes receiving semaglutide 2.4 mg weekly lost on average 14.9% of their baseline body weight by week 68 versus 2.4% with placebo, a treatment difference of 12.4 percentage points [63]. Sustained results were observed in the STEP 5 trial, where at week 104 the semaglutide group had lost 15.2% compared to 2.6% with placebo, a 12.6-percentage-point difference [64]. Across multiple STEP trials, between 69% and 79% of participants reached ≥ 10% weight loss, and a substantial proportion achieved ≥ 15% [63]. The safety profile was consistent with gastrointestinal side effects and without increased hypoglycemia risk [63].

Phentermine/topiramate extended-release has also shown robust results: in the CONQUER trial, the combination at 15 mg/92 mg daily produced 9.8% mean placebo-subtracted weight reduction after 56 weeks, while 7.8% was achieved with the lower 7.5 mg/46 mg dose [65]. In the EQUIP trial among patients with BMI ≥ 35 kg/m^2^, a mean weight loss of 10.9% was observed with the higher dose versus 1.6% with placebo at one year; similar reductions occurred with the lower dose (5.1%) [65]. A network meta-analysis confirmed that phentermine/topiramate had the highest odds of achieving ≥ 5% and ≥10% weight loss versus placebo and versus other agents, including liraglutide, naltrexone–bupropion, and orlistat. However, caution is advised due to psychological side effects and limited approval in some jurisdictions [66].

Orlistat, a pancreatic lipase inhibitor, yields modest benefits: long-term trials report approximately 2.6–3 kg greater weight loss than placebo, and around 35–55% of subjects achieve ≥ 5% weight loss, with 16–25% achieving ≥ 10% [66]. Liraglutide 3.0 mg daily, another GLP-1 receptor agonist, produces an average placebo-adjusted loss of 4–5% weight [67]. Naltrexone-bupropion yields an average excess weight loss of around 5% [67]. Emerging dual agonists such as tirzepatide have shown higher placebo-adjusted weight reductions (~18%) in recent trials, though full obesity approvals and long-term data remain limited within the last ten years [68]. Table 3 provides a comparative overview of anti-obesity medications, including mechanisms of action, dosage, and mean placebo-subtracted weight loss in trials of at least one year.

This comparative overview underscores the importance of tailoring pharmacotherapeutic strategies to individual profiles, taking into account efficacy, safety, comorbidity burden, and adherence potential over time.

#### 3.3.3. Surgical Management

Bariatric surgery remains a crucial therapeutic option in long-term obesity care, particularly for individuals with severe obesity who have not achieved sustained outcomes through lifestyle or pharmacological treatment. According to established clinical guidelines, surgical interventions are typically considered for patients with advanced stages of obesity, especially when cardiometabolic comorbidities are present [69].

Commonly performed procedures include Roux-en-Y gastric bypass (RYGB), sleeve gastrectomy (SG), adjustable gastric banding (AGB), and biliopancreatic diversion with duodenal switch (BPD-DS). These procedures differ in anatomical mechanisms: RYGB combines restriction and malabsorption, SG reduces gastric volume, AGB applies mechanical limitation of intake, and BPD-DS induces substantial nutrient malabsorption. Among them, RYGB and SG are associated with the most favorable outcomes in terms of weight loss and metabolic improvement. In contrast, AGB shows lower long-term efficacy, while BPD-DS, despite its strong metabolic effects, carries an increased risk of nutritional complications and surgical morbidity [70].

Although body mass index remains a key eligibility criterion in surgical protocols, its use should be interpreted in the context of clinical severity and patient-specific health risks.

#### 3.3.4. Metabolic Impact and Comparative Effectiveness

Recent evidence underscores that bariatric surgery is not merely a mechanical intervention but also initiates significant metabolic and hormonal adaptations. These include improvements in insulin sensitivity, modulation of gut hormone secretion, and reductions in systemic inflammation, all of which contribute to type 2 diabetes remission and reduced cardiovascular risk [71]. A broad umbrella review confirmed the association between bariatric surgery and decreased incidence of type 2 diabetes, cancer, and cardiovascular events, highlighting its systemic health benefits [72,73].

Several high-quality meta-analyses provide comparative insights into the two most frequently performed procedures: Roux-en-Y gastric bypass (RYGB) and sleeve gastrectomy (SG). According to Han et al. [70], RYGB results in significantly greater excess weight loss at 12 months compared to SG and demonstrates superior effectiveness in the remission of comorbidities such as type 2 diabetes and dyslipidemia. Similarly, Lei et al. [74] report that RYGB is associated with improved metabolic outcomes and a lower risk of long-term reoperation compared to SG. Additional support for these comparative outcomes comes from a recent meta-analysis of studies published between 2013 and 2023, which confirmed that RYGB yields greater excess weight loss, improved glycemic control, and lower reoperation rates compared to SG. However, it also found that RYGB is more frequently associated with early postoperative complications such as internal hernias and anastomotic ulcers, while SG shows a higher incidence of new-onset gastroesophageal reflux disease and persistent nutritional deficiencies [75].

However, these benefits must be weighed against certain disadvantages. RYGB is associated with a higher overall complication rate and more frequent nutritional deficiencies, including deficiencies in iron, vitamin B12, and folate. Additionally, dumping syndrome occurs more frequently after RYGB. In contrast, SG, while generally safer in terms of short-term complications and nutritional risks, is consistently associated with a significantly increased incidence of de novo gastroesophageal reflux disease (GERD), likely due to anatomical alterations that reduce lower esophageal sphincter pressure and increase intragastric pressure. This adverse effect is notably less prevalent after RYGB, which bypasses the gastroesophageal junction [76].

A structured comparison of clinical outcomes between RYGB and SG is presented in Table 4. For the full comparative data, see Appendix A Table A1. This synthesis, based on two high-quality meta-analyses [74,76], confirms that RYGB generally achieves superior outcomes in terms of excess weight loss, type 2 diabetes and dyslipidemia remission, and lower rates of reoperation. However, this comes at the cost of increased postoperative complications and nutritional deficiencies. SG is associated with fewer general complications, yet shows a significantly higher risk of developing de novo GERD. All reported differences were found to be both statistically significant and clinically meaningful. This table offers a qualitative comparison, emphasizing the direction of effect, statistical significance, and clinical relevance, rather than reporting raw numerical values.

Expanding the indications for bariatric surgery to include individuals with class 1 obesity (BMI 30–35 kg/m^2^) has gained growing support, particularly in cases with obesity-related comorbidities such as type 2 diabetes, hypertension, or metabolic syndrome. According to Yerdel and Özgen [71], patients within this BMI range, especially those of Asian ethnicity or with central adiposity, may derive significant metabolic benefits from surgery despite a lower BMI. Although long-term mortality data are limited for this group, short- and medium-term evidence suggests meaningful improvements in body weight, glycemic control, and quality of life. Notably, sleeve gastrectomy appears to be the preferred option in this population due to its favorable safety profile. These findings underscore the rationale for a more individualized, risk-based approach to surgical eligibility, rather than relying solely on BMI thresholds.

Despite its effectiveness, bariatric surgery carries long-term risks and must be supported by structured follow-up. Common complications include micronutrient deficiencies (iron, vitamin B12, vitamin D, and folate), gastrointestinal issues, and psychological challenges. Thus, comprehensive care involving supplementation protocols, dietary monitoring, and mental health support remains critical [77,78].

Mental health outcomes following bariatric surgery are complex and exhibit both positive and adverse patterns. An umbrella review of 15 meta-analyses demonstrated a moderate and statistically significant reduction in symptoms of depression (SMD ≈ −0.5) and anxiety, particularly during the first 12 to 24 months postoperatively. Patients also reported enhanced self-esteem and quality of life in this period. However, a significant increase in the risk of suicidal ideation and self-harm has been observed in several cohorts, especially following Roux-en-Y gastric bypass. These findings underscore the importance of ongoing psychological evaluation, preoperative screening, and long-term psychosocial support in bariatric care [79].

Despite its efficacy, surgical management is sometimes not pursued in eligible patients. Barriers may include regional disparities in access to care, insurance coverage constraints, and persistent stigma associated with obesity and its treatment. Furthermore, insufficient health literacy and widespread misconceptions about bariatric procedures may influence patients’ decisions and expectations [76].

Although bariatric surgery has long been included in international guidelines for obesity treatment, its long-term success requires embedding it within a chronic care framework. This includes structured preoperative education, coordinated postoperative follow-up, nutritional and psychological support, and ongoing disease monitoring to ensure sustainable outcomes [69,78]. While bariatric surgery offers substantial benefits, long-term outcomes are not determined solely by anatomical or metabolic mechanisms. Psychological factors, patient beliefs, and structural barriers such as access to care or continuity of follow-up play an equally critical role in sustaining treatment success.

### 3.4. Psychosocial Dimensions

Although pharmacological and surgical strategies remain central to weight management, their long-term effectiveness is closely linked to the broader psychosocial environment in which treatment is delivered [1,2]. Beyond metabolic targets, patient outcomes are shaped by psychological resilience, prior experiences with healthcare, perceived legitimacy of treatment, and family support systems [3,4]. This section therefore shifts focus from biological interventions to the relational and subjective factors that determine engagement, adherence, and sustainability of care [3,5]. Obesity must be understood not only as a physiological state but as a condition lived through layers of emotional, social, and interpersonal meaning [1,12]. Addressing these dimensions is key to building equitable and person-centered models of treatment [4,12].

#### 3.4.1. Psychological Impact of Body Weight Stigma

Body weight stigma constitutes a substantial psychosocial burden for individuals with overweight and obesity, often resulting in measurable harm to mental health and quality of life. Approximately 40% of adults report experiencing weight-related stigma, which is independently associated with a 2.5-fold increase in the risk of mood and anxiety disorders, even after adjusting for BMI and other factors [14]. Internalized weight bias—reflected in the personal acceptance of societal stereotypes—has been linked to reduced self-efficacy and maladaptive behaviors. In clinical populations, it is reported by over half of individuals with obesity [14].

Psychological consequences of stigma can impair treatment adherence and reduce motivation for health-promoting behaviors. In a study conducted at a multidisciplinary bariatric clinic in India (n = 146), higher stigma scores were significantly associated with dysfunctional eating patterns and the presence of psychiatric diagnoses (adjusted odds ratios 3.87 and 3.00, respectively; *p* < 0.05) [13]. These findings underscore the need to integrate stigma-reduction strategies and routine psychiatric screening into all stages of obesity management [13,14].

#### 3.4.2. Psychosocial Origins, Stigma, and Clinical Implications

Although the primary focus of this review is adult obesity, growing evidence shows that early life experiences critically shape long-term behavioral and psychological trajectories. Adverse childhood experiences (ACEs)—including abuse, neglect, and household dysfunction—have been consistently associated with an elevated risk of obesity, especially when multiple adversities co-occur. A systematic review found a significant link between ACEs and childhood obesity, underscoring how early-life vulnerabilities can disrupt self-regulation and contribute to maladaptive eating behaviors that track into adult life [80].

Meta-analyses demonstrate that adults exposed to multiple ACEs have a 46% higher risk of developing obesity compared to those without such histories, as reported by Wiss and Brewerton (2020), while Zhou et al. (2024) found a 48% higher risk [81,82]. Mechanistically, this association is mediated by chronic stress activation, impaired emotion regulation, and reliance on maladaptive coping strategies—particularly those involving food. Zhou et al. also highlight notable sex-specific effects, with women showing heightened vulnerability to the obesogenic consequences of early trauma.

Family dynamics—including parental modeling of dieting behavior, emotional feeding, and weight-based teasing—can reinforce internalized body weight stigma from an early age. Such stigma is often compounded by peer interactions and social environments, such as bullying, exclusion, or appearance-related criticism, laying the foundation for dysfunctional relationships with food and persistent shame into adulthood [5,81]. This developmental trajectory may be especially pronounced in individuals with comorbid psychiatric conditions. It has been reported that adults with severe mental illness and a history of ACEs are disproportionately affected by metabolic disturbances, including obesity, suggesting an additive burden of psychosocial vulnerability [83].

In parallel, longitudinal cohort studies confirm that childhood obesity itself strongly predicts obesity in adulthood. According to Simmonds et al. (2016), children with obesity are approximately five times more likely to remain obese later in life, with more than 70% of obese adolescents maintaining their obesity into their thirties [84]. These findings emphasize the long-term imprint of early adversities and the urgent need for preventive and reparative intervention strategies.

From a clinical perspective, internalized stigma resulting from early weight-based criticism can manifest as healthcare avoidance in adulthood. Individuals who have experienced teasing, shaming, or judgment about their body weight in childhood often report fear or mistrust toward medical professionals. This avoidance pattern resembles other forms of internalized stigma—such as that seen in mental health contexts—where shame impedes timely access to care and worsens health outcomes [85].

Given the pervasive role of psychosocial factors in obesity, it is imperative to incorporate stigma-aware assessments into routine care. Body weight stigma is now recognized as a determinant of both the etiology and persistence of obesity, influencing behaviors such as disordered eating, physical inactivity, and disengagement from healthcare systems [3]. To address these barriers, clinical evaluations should include a comprehensive body mass- and stigma-informed history, examining factors such as internalized bias, mental health status, and social determinants of health. Standardized psychosocial assessment tools may help identify individuals in need of multidisciplinary support—particularly psychological or behavioral interventions—which are critical for long-term adherence and recovery [3].

Furthermore, stigmatizing experiences can undermine the effectiveness of both lifestyle and pharmacological interventions. Individuals who feel judged or ashamed because of their body weight are more likely to drop out of treatment programs, avoid physical activity, or discontinue medications such as GLP-1 receptor agonists [86]. As such, treatment strategies must integrate stigma-reduction approaches, behavioral counseling, and positive identity reinforcement. Trauma-informed care, motivational interviewing, and non-stigmatizing communication can foster trust, improve adherence, and enhance therapeutic outcomes. Several national healthcare systems have responded to this evidence by integrating psychosocial care into their obesity management frameworks. Multidisciplinary models that combine behavioral therapy, psychological counseling, and community-based support programs have been implemented, for example, in Canada, the United Kingdom, and Australia. In Canada, the integration of psychosocial care within obesity treatment frameworks has improved patient engagement and adherence to lifestyle changes, leading to better long-term weight management outcomes and improved quality of life [87,88]. In the UK, multidisciplinary weight management services that include psychological assessment and support have been shown to reduce weight stigma experienced by patients both in healthcare settings and the workplace, which contributes to improved mental health and social functioning [89,90]. Addressing psychosocial factors also mitigates obesity-related stigma in society and the workplace, which is linked to adverse effects such as reduced employment opportunities, discrimination, and decreased self-esteem [53]. Psychosocial interventions, including cognitive-behavioral therapy (CBT) and motivational interviewing, help patients develop coping strategies and reduce internalized stigma, thereby improving treatment adherence and functional capacity [91,92]. In comparison, obesity treatments that do not include psychological components tend to focus primarily on physical health outcomes, such as weight reduction and metabolic parameters. These approaches may underestimate the influence of mental health on eating behaviors, motivation, and social barriers, potentially limiting long-term success. Integrating psychosocial care creates a more holistic treatment paradigm that addresses the complex biopsychosocial nature of obesity [90].

Taken together, these psychosocial dimensions highlight the need for a paradigm shift in obesity care—one that centers empathy, psychological safety, and long-term patient engagement. Healthcare systems must be equipped to address not only current stressors but also the enduring impact of early-life stigma. This includes training clinicians in stigma-sensitive practices, implementing trauma-informed protocols, and integrating psychosocial screening into obesity management pathways. Ultimately, reducing stigma-driven avoidance and promoting dignity-centered care are essential to improving health outcomes and quality of life in people living with obesity.

## 4. Strengths and Limitations of the Study

This study provides a comprehensive and interdisciplinary synthesis of current evidence on the clinical, psychosocial, and therapeutic dimensions of obesity. One of its primary strengths lies in the integration of recent international guidelines, pharmacological developments, and psychosocial insights, offering a multifaceted understanding of obesity as a complex, chronic disease. By drawing upon literature from diverse fields—including endocrinology, psychiatry, public health, and epidemiology—this review contributes to the ongoing reframing of obesity care beyond simplistic weight-centric models. Additionally, the paper critically engages with the evolving role of diagnostic frameworks and highlights the clinical implications of weight stigma, which is often underrepresented in scientific discourse. The inclusion of both biomedical and sociocultural perspectives provides a nuanced lens that may help inform more effective and inclusive approaches to diagnosis, treatment, and health policy. However, several limitations must be acknowledged. First, as a narrative review, the study is inherently selective and subject to potential bias in the literature included. Despite efforts to prioritize high-quality, peer-reviewed sources from the past decade, the review may not capture the full range of emerging perspectives or unpublished data. Second, most of the cited evidence originates from high-income countries, which may limit generalizability to underrepresented populations with differing healthcare systems and sociocultural dynamics. Furthermore, while recent pharmacotherapies are discussed, real-world long-term data on their safety, accessibility, and adherence remain limited. The integration of psychosocial variables, though emphasized, is still constrained by a lack of standardized diagnostic and assessment tools across studies. Finally, although the review touches on structural determinants of health, further empirical research is needed to operationalize and address these factors in clinical practice. Future research should focus on longitudinal outcomes of integrated treatment models, cross-cultural validation of diagnostic frameworks, and the development of scalable, stigma-informed interventions in both clinical and community settings.

## 5. Conclusions

This review highlights the evolving recognition of obesity as a chronic, multifactorial disease requiring a multidimensional approach to diagnosis and treatment. Advances in diagnostic tools now emphasize functional impact, adiposity distribution, and metabolic risk, moving beyond BMI [3,35,38]. While lifestyle interventions remain foundational, their long-term success depends on integrating biological, psychological, and social factors [1,4,14]. Pharmacotherapies, particularly GLP-1 receptor agonists, have shown efficacy but vary in safety and accessibility [9,59,93]. The need to address psychological burdens, such as weight-related stigma and co-occurring psychiatric conditions, is crucial for improving treatment adherence [13,14,59]. This review supports a comprehensive, integrated care model that incorporates these aspects, as outlined in Table 5, which summarizes the most important aspects in the advancement of obesity treatment, to ensure more sustainable and equitable outcomes in obesity management [3,57].

## Figures and Tables

**Table 1:** Definitions of obesity by leading institutions.

Source	Year	Definition	Key Implications
World Health Organization (WHO) [31]	2000–present	Obesity is defined as abnormal or excessive fat accumulation that may impair health, typically measured by BMI ≥ 30.	Uses BMI as the main diagnostic threshold. Acknowledges its limitations, such as failing to account for fat distribution or muscle mass.
ICD-11 (WHO) [32]	2022	Obesity is a chronic disease characterized by abnormal or excessive fat accumulation that presents a risk to health.	Classified under code 5B81 as a disease of energy homeostasis dysregulation. Highlights systemic and metabolic consequences.
The Lancet Commission [33]	2023	Clinical obesity is defined by health impairments and functional consequences caused by excess or dysfunctional fat, not solely by body weight.	Calls for moving beyond BMI; introduces terms like ‘preclinical’ and ‘clinical obesity’ based on physiological impairment.
Rubino et al. [3]	2025	Obesity is a disease characterized by abnormal or excessive adipose tissue that impairs health and physiological function.	Proposes a broader biological framework, focusing on adipose dysfunction and systemic effects. Criticized by EASO for lacking operational diagnostic criteria.
European Association for the Study of Obesity (EASO) [20]	2025	Obesity is a complex, relapsing, and chronic disease in which excess or dysfunctional adiposity impairs health.	Obesity is a complex, relapsing, and chronic disease in which excess or dysfunctional adiposity impairs health.
Obesity Canada [4]	2023	Obesity is a chronic disease characterized by excess or dysfunctional adiposity that impairs health and well-being.	Emphasizes patient-centered care and the importance of reducing internalized stigma. Highlights the role of psychological and behavioral factors.
Korean Society for the Study of Obesity (KSSO) [19]	2023	Obesity is a chronic disease involving excessive body fat that negatively affects health, with diagnostic criteria adapted to Asian populations.	Recommends ethnicity-specific BMI cutoffs. Promotes phenotypic and functional approaches to diagnosis.
American Association of Clinical Endocrinology (AACE) [34]	2023	Obesity is a chronic disease known as Adiposity-Based Chronic Disease (ABCD), reflecting both excess fat mass and its clinical complications.	Introduces the ABCD model to improve staging and personalize treatment. Stresses the need for reducing diagnostic and management bias.

While Rubino et al. [3] is not an institutional guideline, it reflects a widely debated expert consensus and has influenced current definitional discourse.

**Table 2:** Diagnostic criteria for obesity.

Diagnostic Criterion	WHO [43]	EOSS [44]	Lancet Diabetes and Endocrinology Commission (2025) [45]
Anthropometric measures (BMI, WC)	Included	Included as initial classification	Included
Body composition (e.g., % body fat)	Not included	Not included	Included
Psychological burden	Not included	Included (stages 1–4)	Indirectly included
Physical comorbidities	Not included	Included (stages 1–4)	Included
Functional capacity (daily limitations)	Not included	Included (stages 2–4)	Included (criterion for clinical obesity)

**Table 3:** Comparison of selected pharmacological treatments for obesity.

Drug/Class	Mechanism/Site of Action	Typical Dose/Duration	Mean Placebo-Subtracted Weight Loss	Source
Semaglutide (GLP1 RA)	GLP1 receptor agonist reduces hunger, slows gastric emptying	2.4 mg SC once weekly, 68–104 weeks	≈12.4% at 68 wk; ≈12.6% at 104 wk	Wilding et al. (2021) [64], Garvey et al. (2022) [65]
Phentermine/topiramate ER	Sympathomimetic + GABA/glutamate modulation, appetite suppression	15/92 mg daily (high dose), ≥56 weeks	≈9.8% (high dose); ≈7.8% (mid dose) vs. placebo	Torgerson et al. (2004) [66]
Liraglutide	GLP1 receptor agonist (daily)	3.0 mg SC daily, ≥1 year	≈4–5% excess loss	Khera et al. (2016) [67]
Naltrexone–bupropion	Opioid antagonist + NE/dopamine reuptake inhibitor	Approved fixed-dose combo, up to 56 weeks	≈5% vs. placebo	Khera et al. (2016) [67]
Orlistat	Inhibits pancreatic lipase, reduces fat absorption	120 mg TID, ≥1 year	≈2.6–3 kg extra loss; 5–10% of patients lose ≥10% body weight	Torgerson et al. (2004) [66]
Tirzepatide (GIP/GLP1 RA)	Dual incretin agonist (GIP + GLP1)	5–15 mg weekly for 72–88 weeks	≈18% placebo-adjusted (in SURMOUNT-1)	Jastreboff et al. (2022) [68]

**Table 4:** Comparative evaluation of RYGB versus SG across selected clinical outcomes.

Clinical Parameter	Superior Outcome	Statistically Significant?	Clinically Meaningful?	Timeframe	Source(s)
Excess Weight Loss (EWL)	RYGB	Yes	Yes	5 years	Lei et al. (2024) [74]
Type 2 Diabetes Remission	RYGB	Yes	Yes	5 years	Lei et al. (2024) [74]
General Postoperative Morbidity	SG	Yes	Yes	5 years	Lei et al. (2024) [74]
Total Complications (RR)	RYGB	Yes	Yes	Midterm–long term	Han et al. (2020) [70]
Reoperation Rate	RYGB	Yes	Yes	Midterm–long term	Han et al. (2020) [70]

Note: This table provides a qualitative synthesis of outcomes—highlighting direction of effect, statistical significance, and clinical relevance—rather than absolute numerical values. The data are based on two high-quality meta-analyses: Han et al. [70] and Lei et al. [74]. A detailed summary is available in the Table A1.

**Table 5:** Summary of the most important aspects in the advancement of obesity treatment.

Domain	Key Advances	Implications for Care	Challenges/Notes
Diagnostic Advances	- From BMI to multifactorial tools: waist circumference, body fat %, visceral fat imaging - Focus on functional impact and metabolic risk - Recognition of obesity as a chronic, relapsing disease	- Improved diagnostic accuracy and risk stratification - Enables personalized, functional assessment-based diagnosis	Need for tools reflecting diverse populations
Treatment Strategies	- Shift from short-term behavioral to integrated, multidisciplinary care - Inclusion of pharmacotherapies (GLP-1 RAs, polyagonists) - Individualized treatment planning	- Enhanced long-term weight management and adherence - Addresses biological and psychosocial components	Variable safety, tolerability, and limited long-term real-world data
Psychosocial Factors	- Recognition of stigma as a major barrier - Integration of mental health support - Psychosocial factors independently affect outcomes - Addressing internalized stigma is critical	- Reduces healthcare avoidance and improves engagement - Fundamental target in treatment models	Persistent stigma in society and healthcare systems
Integrated Approach	- Interdependence of diagnosis, treatment, and psychosocial care - Multidisciplinary, patient-centered management - Chronic disease framework	- Holistic care addressing biological, psychological, and social determinants - Sustainable, equitable outcomes	Research gaps on diverse populations and long-term interventions

## Data Availability

Not applicable.

## Abbreviations

The following abbreviations are used in this manuscript:

BMI	Body Mass Index
GLP-1 RA	Glucagon-Like Peptide-1 Receptor Agonist
RYGB	Roux-en-Y Gastric Bypass
SG	Sleeve Gastrectomy
MASLD	Metabolic Dysfunction-Associated Steatotic Liver Disease
EOSS	Edmonton Obesity Staging System
ACEs	Adverse childhood experiences
AACE	American Association of Clinical Endocrinology
DEXA	Dual-Energy X-ray Absorptiometry
WHO	World Health Organization

## Appendix A

**Table A1:** Comparative outcomes of Roux-en-Y gastric bypass (RYGB) versus sleeve gastrectomy (SG).

Clinical Parameter	Superior Outcome	Statistical Significance	Clinical Meaningfulness	Timeframe	Source(s)
Excess Weight Loss (EWL)	RYGB	Yes	Yes	5 years	Lei et al. (2024) [74]
Type 2 Diabetes Remission	RYGB	Yes	Yes	5 years	Lei et al. (2024) [74]
Hypertension Remission	No difference	No	No	5 years	Lei et al. (2024) [74]
Dyslipidemia Remission	RYGB	Yes	Yes	5 years/~3 years	Lei et al. (2024), Han (2020) [70,74]
Obstructive Sleep Apnea Remission	RYGB	Yes	Yes	5 years	Lei et al. (2024) [74]
Quality of Life (GIQLI)	No difference	No	No	5 years	Lei et al. (2024) [74]
General Postoperative Morbidity	SG	Yes	Yes	5 years	Lei et al. (2024) [74]
Total Complications (RR)	RYGB	Yes	Yes	Midterm–long term	Han et al. (2020) [70]
Reoperation Rate	RYGB	Yes	Yes	Midterm–long term	Han et al. (2020) [70]
De novo GERD	SG worse	Yes	Yes	Midterm–long term	Han et al. (2020) [70]
Dumping Syndrome	RYGB worse	Yes	Yes	Midterm–long term	Han et al. (2020) [70]

## References

[1] Apovian C.M., Guo X.-R., Hawley J.A., Karmali S., Loos R.J.F., Waterlander W.E. (2023). Approaches to Addressing the Rise in Obesity Levels. Nat. Rev. Endocrinol..

[2] Lobstein T., Jackson-Leach R., Powis J., Brinsden H., Gray M. (2023). World Obesity Atlas 2023.

[3] Rubino F., Cummings D.E., Eckel R.H., Cohen R.V., Wilding J.P.H., Brown W.A., Batterham R.L., Farooqi I.S., Farpour-Lambert N.J., Roux C.W.L. (2025). Definition and Diagnostic Criteria of Clinical Obesity. Lancet Diabetes Endocrinol..

[4] English S., Vallis M. (2023). Moving Beyond Eat Less, Move More Using Willpower: Reframing Obesity as a Chronic Disease—Impact of the 2020 Canadian Obesity Guidelines Reframed Narrative on Perceptions of Self and the Patient–Provider Relationship. Clin. Obes..

[5] Andersen K.K. (2022). Examining Memorable Weight Stigma Messages and the Effects on Weight-Related Outcomes. Ph.D. Thesis.

[6] World Health Organization (WHO) (2022). European Regional Obesity Report 2022.

[7] Stival C., Lugo A., Odone A., van den Brandt P.A., Fernandez E., Tigova O., Soriano J.B., López M.J., Scaglioni S., Gallus S. (2022). Prevalence and Correlates of Overweight and Obesity in 12 European Countries in 2017-2018. Obes. Facts..

[8] Koliaki C., Dalamaga M., Liatis S. (2023). Update on the Obesity Epidemic: After the Sudden Rise, Is the Upward Trajectory Beginning to Flatten?. Curr. Obes. Rep..

[9] Govender I., Sunnasy A. (2025). The Growing Problem of Obesity in South Africa. S. Afr. Fam. Pract..

[10] Bansal D., Mohammed S.V.S., Devi N., Boya C., Dhora Babu K., Dutta P. (2024). Trends Estimation of Obesity Prevalence among South Asian Young Population: A Systematic Review and Meta-Analysis. Sci. Rep..

[11] Tham K.W., Abdul Ghani R., Cua S.C., Deerochanawong C., Fojas M., Hocking S., Lee J., Nam T.Q., Pathan F., Saboo B. (2023). Obesity in South and Southeast Asia-A New Consensus on Care and Management. Obes. Rev..

[12] Donini L.M., Pinto A., Giusti A.M., Lenzi A., Poggiogalle E. (2020). Obesity or BMI Paradox? Beneath the Tip of the Iceberg. Front. Nutr..

[13] Jiwanmall S.A., Kattula D., Nandyal M.B., Parvathareddy S., Kirubakaran R., Jebasingh F., Paul T.V., Thomas N.J., Kapoor N. (2022). Weight Stigma in Patients with Obesity and Its Clinical Correlates: A Perspective from an Indian Bariatric Clinic. Cureus.

[14] Kirk S.F.L., Ramos Salas X., Alberga A.S., Russell-Mayhew S. (2020). Reducing Weight Bias in Obesity Management, Practice and Policy.

[15] Jiang Y., Zhu H., Gong F. (2024). Why Does GLP-1 Agonist Combined with GIP and/or GCG Agonist Have Greater Weight Loss Effect Than GLP-1 Agonist Alone in Obese Adults Without Type 2 Diabetes?. Diabetes Obes. Metab..

[16] Ahiwale R.J., Dasari P., Nevis I. (2024). Glucagon-like Peptide-1 (GLP-1) Receptor Agonists—Efficacy, Safety, and Cost-Effectiveness in Obesity Management: A Systematic Review. Value Health.

[17] Drame M., Godaert L. (2023). The Obesity Paradox and Mortality in Older Adults: A Systematic Review. Nutrients.

[18] Ferrari R. (2015). Writing Narrative Style Literature Reviews. Med. Writ..

[19] Haam J.H., Kim B.T., Kim E.M., Kwon H., Kang J.H., Park J.H., Kim K.-K., Rhee S.Y., Kim Y.-H., Lee K.Y. (2023). Diagnosis of Obesity: 2022 Update of Clinical Practice Guidelines for Obesity by the Korean Society for the Study of Obesity. J. Obes. Metab. Syndr..

[20] European Association for the Study of Obesity (EASO) (2025). EASO response to the Lancet Commission report on obesity diagnosis and management. https://easo.org/easo-response-to-the-lancet-commission-report-on-obesity-diagnosis-and-management/.

[21] Stein D.J., Szatmari P., Gaebel W., Berk M., Vieta E., Maj M., de Vries Y.A., Roest A.M., de Jonge P., Maercker A. (2020). Mental, Behavioral and Neurodevelopmental Disorders in the ICD-11: An International Perspective on Key Changes and Controversies. BMC Med..

[22] Qian Y., Han Y., Liu D., Wang X., Jin X., Sun X. (2025). Association between Body Composition Components and Bone Mineral Density in Older Adults. Sci. Rep..

[23] Wu Y., Li D., Vermund S.H. (2024). Advantages and Limitations of the Body Mass Index (BMI) to Assess Adult Obesity. Int. J. Environ. Res. Public. Health..

[24] Byker Shanks C., Bruening M., Yaroch A.L. (2025). BMI or Not to BMI? Debating the Value of Body Mass Index as a Measure of Health in Adults. Int. J. Behav. Nutr. Phys. Act..

[25] Heymsfield S.B., Ramirez S., Yang S., Thomas D.M., Brown J.C., Compton S.L.E., Schuna J.M., Smith S.R., Ludwig D.S., Ebbeling C.B. (2025). Critical Analysis of Dual-Energy X-ray Absorptiometry-Measured Body Composition Changes with Voluntary Weight Loss. Obesity..

[26] Sweatt K., Garvey W.T., Martins C. (2024). Strengths and Limitations of BMI in the Diagnosis of Obesity: What Is the Path Forward?. Curr. Obes. Rep..

[27] Muntean P., Miclos-Balica M., Macavei G.A., Munteanu O., Neagu A., Neagu M. (2024). Anthropometric Formulas Repurposed to Predict Body Fat Content from Ultrasound Measurements of Subcutaneous Fat Thickness. Symmetry.

[28] Jeong S.-M., Lee D.H., Rezende L.F.M., Giovannucci E.L. (2023). Different Correlation of Body Mass Index with Body Fatness and Obesity-Related Biomarkers According to Age, Sex and Race-Ethnicity. Sci. Rep..

[29] Čermáková E., Forejt M. (2024). Metabolically Healthy Obesity and Health Risks—A Review of Meta-Analyses. Cent. Eur. J. Public. Health.

[30] Elkan M., Kofman N., Minha S., Rappoport N., Zaidenstein R., Koren R. (2023). Does the “Obesity Paradox” Have an Expiration Date? A Retrospective Cohort Study. J. Clin. Med..

[31] World Health Organization (WHO) (2000). Obesity: Preventing and Managing the Global Epidemic. Report of a WHO Consultation on Obesity.

[32] World Health Organization (WHO) (2022). ICD-11 for Mortality and Morbidity Statistics: 5B81 Obesity. https://icd.who.int/browse/2025-01/mms/en#149403041.

[33] Rubino F., Batterham R.L., Koch M., Mingrone G., le Roux C.W., Farooqi I.S., Farpour-Lambert N., Gregg E.W., Cummings D.E. (2023). Lancet Diabetes & Endocrinology Commission on the Definition and Diagnosis of Clinical Obesity. Lancet Diabetes Endocrinol..

[34] Nadolsky K., Addison B., Agarwal M., Almandoz J.P., Bird M.D., Chaplin M.D., Garvey W.T., Kyle T.K. (2023). American Association of Clinical Endocrinology Consensus Statement: Addressing Stigma and Bias in the Diagnosis and Management of Patients with Obesity/Adiposity-Based Chronic Disease and Assessing Bias and Stigmatization as Determinants of Disease Severity. Endocr. Pract..

[35] Purnell J.Q. (2023). What Is Obesity?: Definition as a Disease, with Implications for Care. Gastroenterol. Clin. N. Am..

[36] Riegel K.D., Konecna J., Matoulek M., Rosova L. (2021). Implementation of the DSM-5 and ICD-11 Dimensional Models of Maladaptive Personality Traits into Pre-Bariatric Assessment. Front. Psychol..

[37] Smolik R., Gaweł M., Kliszczyk D., Sasin N., Szewczyk K., Górnicka M. (2025). Comparative Analysis of Body Composition Results Obtained by Air Displacement Plethysmography (ADP) and Bioelectrical Impedance Analysis (BIA) in Adults. Appl. Sci..

[38] Potter A.W., Chin G.C., Looney D.P., Friedl E.K. (2025). Defining Overweight and Obesity by Percent Body Fat Instead of Body Mass Index. J. Clin. Endocrinol. Metab..

[39] Fitch A.K., Bays H.E. (2022). Obesity Definition, Diagnosis, Bias, Standard Operating Procedures (SOPs), and Telehealth: An Obesity Medicine Association (OMA) Clinical Practice Statement (CPS) 2022. Obes. Pillars.

[40] Cao B., Xu J., Li R., Teopiz K.M., McIntyre R.S., Chen H. (2022). Interventions targeting comorbid depression and overweight/obesity: A systematic review. J. Affect. Disord..

[41] Pilati I., Slee A., Frost R. (2022). Sarcopenic Obesity and Depression: A Systematic Review. J. Frailty Aging.

[42] Abiri B., Hosseinpanah F., Banihashem S., Madinehzad S.A., Valizadeh M. (2022). Mental health and quality of life in different obesity phenotypes: A systematic review. Health Qual. Life Outcomes.

[43] World Health Organization Obesity and Overweight. https://www.who.int/news-room/fact-sheets/detail/obesity-and-overweight.

[44] Sharma A.M., Kushner R.F. (2009). A Proposed Clinical Staging System for Obesity. Int. J. Obes..

[45] Blüher M., Frühbeck G., Oppert J.M., Halford J., Dicker D., Yumuk V., Müller-Stierlin A., Busetto L. (2025). Obesity: A New Definition and Framework for Care. Lancet Diabetes Endocrinol..

[46] Lu X., Jin Y., Li D., Zhang J., Han J., Li Y. (2022). Multidisciplinary Progress in Obesity Research. Genes.

[47] Van Baak M.A., Mariman E.C.M. (2023). Obesity-Induced and Weight-Loss-Induced Physiological Factors Affecting Weight Regain. Nat. Rev. Endocrinol..

[48] Machado A.M., Guimarães N.S., Bocardi V.B., da Silva T.P.R., Carmo A.S.D., Menezes M.C., Duarte C.K. (2022). Understanding Weight Regain after a Nutritional Weight Loss Intervention: Systematic Review and Meta-Analysis. Clin. Nutr. ESPEN..

[49] Van Baak M.A., Mariman E.C.M. (2025). Physiology of Weight Regain after Weight Loss: Latest Insights. Curr. Obes. Rep..

[50] Nutter S., Eggerichs L.A., Nagpal T.S., Ramos Salas X., Chea C.C., Saiful S., Ralston J., Barata-Cavalcanti O., Batz C., Baur L.A. (2023). Changing the Global Obesity Narrative to Recognize and Reduce Weight Stigma: A Position Statement from the World Obesity Federation. Obes. Rev..

[51] Madsen M., Michaelsen L., DeCosta P., Grabowski D. (2024). Stigma-Generating Mechanisms in Families Enrolled in a Pediatric Weight Management Program: A Qualitative Study of Health Identities and Healthcare Authenticity. Children.

[52] Cassar K. (2023). Factors Influencing Physical Activity Among Diabetics in Malta and Gozo. Masters’ Thesis.

[53] Rubino F., Puhl R.M., Cummings D.E., Eckel R.H., Ryan D.H., Mechanick J.I., Nadglowski J., Ramos Salas X., Schauer P.R., Twenefour D. (2020). Joint International Consensus Statement for Ending Stigma of Obesity. Nat. Med..

[54] Moll H., Frey E., Gerber P., Geidl B., Kaufmann M., Braun J., Beuschlein F., Puhan M.A., Yebyo H.G. (2024). GLP-1 Receptor Agonists for Weight Reduction in People Living with Obesity but without Diabetes: A Living Benefit-Harm Modelling Study. EClinicalMedicine.

[55] Rodrigues Silva Sombra L., Anastasopoulou C. (2025). Pharmacologic Therapy for Obesity.

[56] O’Shea D., Kahan S., Lennon L., Breen C. (2021). Practical Approaches to Treating Obesity: Patient and Healthcare Professional Perspectives. Adv. Ther..

[57] Yin Y., Zhang M., Cao Q., Lin L., Lu J., Bi Y., Chen Y. (2025). Efficacy of GLP-1 Receptor Agonist-Based Therapies on Cardiovascular Events and Cardiometabolic Parameters in Obese Individuals Without Diabetes: A Meta-Analysis of Randomized Controlled Trials. J. Diabetes..

[58] Badve S.V., Bilal A., Lee M.M.Y., Sattar N., Gerstein H.C., Ruff C.T., McMurray J.J.V., Rossing P., Bakris G., Mahaffey K.W. (2025). Effects of GLP-1 receptor agonists on kidney and cardiovascular disease outcomes: A meta-analysis of randomised controlled trials. Lancet Diabetes Endocrinol..

[59] Kornelius E., Huang J.Y., Lo S.C., Huang C.N., Yang Y.S. (2024). The risk of depression, anxiety, and suicidal behavior in patients with obesity on glucagon like peptide-1 receptor agonist therapy. Sci. Rep..

[60] Arillotta D., Floresta G., Guirguis A., Corkery J.M., Catalani V., Martinotti G., Sensi S.L., Schifano F. (2023). GLP-1 Receptor Agonists and Related Mental Health Issues; Insights from a Range of Social Media Platforms Using a Mixed-Methods Approach. Brain Sci..

[61] Vandvik P.O., Nagraj S.K., Agarwal A., Agoritsas T., Li S. (2025). Proposal for the Addition of Glucagon-Like Peptide-1 (GLP-1) Receptor Agonists to the WHO Model List of Essential Medi-cines for the Treatment of Adults with Obesity. MAGIC Evidence Ecosystem Foundation. https://cdn.who.int/media/docs/default-source/2025-eml-expert-committee/addition-of-new-medicines/a.14_glp-1-obesity.pdf.

[62] Yue J., Wang Q., Yu A.X., Wei Y., Wei Y., Dong-Rui L., Jin J.-Y., Zhang H.-C., Zhang X.-F. (2023). GLP-1 Receptor Agonists for the Treatment of Obesity: Role as a Promising Approach. Front. Endocrinol..

[63] Jaison K.I., Asharaf H., Thimothy G., Jose J., Paily R., Josey J., Sajna S., Radhakrishnan R. (2024). Psychological Impact of Obesity: A Comprehensive Analysis of Health-Related Quality of Life and Weight-Related Symptoms. Obes. Med..

[64] Wilding J.P.H., Batterham R.L., Calanna S., Davies M., Van Gaal L.F., Lingvay I., McGowan B.M., Rosenstock J., Tran M.T., Wadden T.A. (2021). Once-Weekly Semaglutide in Adults with Overweight or Obesity. N. Engl. J. Med..

[65] Garvey W.T., Batterham R.L., Bhatta M., Buscemi S., Christensen L.N., Frias J.P., Jódar E., Kandler K., Rigas G., Wadden T.A. (2022). Two-Year Effects of Semaglutide in Adults with Overweight or Obesity: The STEP 5 Trial. Nat. Med..

[66] Torgerson J.S., Hauptman J., Boldrin M.N., Sjöström L. (2004). XENical in the Prevention of Diabetes in Obese Subjects (XENDOS) Study; EQUIP and CONQUER Trials of Phentermine/Topiramate Extended Release in Obesity. Diabetes Care..

[67] Khera R., Murad M.H., Chandar A.K., Dulai P.S., Wang Z., Prokop L.J., Loomba R., Camilleri M., Singh S. (2016). Association of Pharmacological Treatments for Obesity with Weight Loss and Adverse Events: A Systematic Review and Meta-Analysis. JAMA.

[68] Jastreboff A.M., Aronne L.J., Ahmad N.N., Wharton S., Connery L., Alves B., Kiyosue A., Zhang S., Liu B., Bunck M.C. (2022). Tirzepatide Once Weekly for the Treatment of Obesity. N. Engl. J. Med..

[69] Kermansaravi M., Valizadeh R., Shahsavan M., Maleknia S.A., Eghbali F., Pazouki A., Shahmiri S.S. (2024). Metabolic and Bariatric Surgery in Patients with Class I Obesity: A Two-Year Follow-Up. BMC Surg..

[70] Han Y., Jia Y., Wang H., Cao L., Zhao Y. (2020). Comparative Analysis of Weight Loss and Resolution of Comorbidities between Laparoscopic Sleeve Gastrectomy and Roux-en-Y Gastric Bypass: A Systematic Review and Meta-Analysis Based on 18 Studies. Int. J. Surg..

[71] Yerdel M.A., Özgen G., Ahmad S.I. (2024). Bariatric Surgery in Class 1 Obesity. Obesity.

[72] Kim M.S., Kim J.Y., Song Y.S., Hong S., Won H., Kim W.J., Kwon Y., Ha J., Fiedorowicz J.G., Solmi M. (2024). Association of Bariatric Surgery with Indicated and Unintended Outcomes: An Umbrella Review and Meta-Analysis for Risk–Benefit Assessment. Obes. Rev..

[73] Wu Z., Gao Z., Qiao Y., Chen F., Guan B., Wu L., Cheng L., Huang S., Yang J. (2023). Long-Term Results of Bariatric Surgery in Adolescents with at Least 5 Years of Follow-Up: A Systematic Review and Meta-Analysis. Obes. Surg..

[74] Lei Y., Lei X., Chen G., Wang Z., Song H., Feng X., Wu Y., Jia V., Hu J., Tian Y. (2024). Update on Comparison of Laparoscopic Sleeve Gastrectomy and Laparoscopic Roux-en-Y Gastric Bypass: A Systematic Review and Meta-Analysis of Weight Loss, Comorbidities, and Quality of Life at 5 Years. BMC Surg..

[75] Kim J.C., Kim M.G., Park J.K., Lee S., Kim J., Cho Y.-S., Kong S.-H., Park D.J., Lee H.-J., Yang H.-K. (2023). Outcomes and Adverse Events after Bariatric Surgery: An Updated Systematic Review and Meta-Analysis, 2013–2023. J. Metab. Bariatr. Surg..

[76] Köhler H., Dorozhkina R., Gruner-Labitzke K., de Zwaan M. (2020). Specific Health Knowledge and Health Literacy of Patients before and after Bariatric Surgery: A Cross-Sectional Study. Obes. Facts.

[77] Stenberg E., dos Reis Falcão L.F., O’Kane M., Liem R., Pournaras D.J., Salminen P., Urman R.D., Wadhwa A., Gustafsson U.O., Thorell A. (2022). Guidelines for Perioperative Care in Bariatric Surgery: Enhanced Recovery After Surgery (ERAS) Society Recommendations: A 2021 Update. World J. Surg..

[78] Gasmi A., Bjørklund G., Mujawdiya P.K., Semenova Y., Peana M., Dosa A., Piscopo S., Gasmi Benahmed A., Costea D.O. (2022). Micronutrients Deficiencies in Patients after Bariatric Surgery. Eur. J. Nutr..

[79] Law S., Dong S., Zhou F., Zheng D., Wang C., Dong Z. (2023). Bariatric Surgery and Mental Health Outcomes: An Umbrella Review. Front. Endocrinol..

[80] Schroeder K., Schuler B.R., Kobulsky J.M., Sarwer D.B. (2021). The Association between Adverse Childhood Experiences and Childhood Obesity: A Systematic Review. Obes. Rev..

[81] Wiss D.A., Brewerton T.D. (2020). Adverse Childhood Experiences and Adult Obesity: A Systematic Review of Plausible Mechanisms and Meta-Analysis of Cross-Sectional Studies. Physiol. Behav..

[82] Zhou Y., Sun Y., Pan Y., Dai Y., Xiao Y., Yu Y. (2024). Association between Adverse Childhood Experiences and Obesity, and Sex Differences: A Systematic Review and Meta-Analysis. J. Psychiatr. Res..

[83] Balaji S., Sankaranarayanan A. (2023). The Association between Adverse Childhood Experiences and Metabolic Syndrome in Severe Mental Illness: A Literature Review. Australas. Psychiatry.

[84] Simmonds M., Llewellyn A., Owen C.G., Woolacott N. (2016). Predicting Adult Obesity from Childhood Obesity: A Systematic Review and Meta-Analysis. Obes. Rev..

[85] Hay P., Aouad P., Le A., Marks P., Maloney D., Touyz S., Maguire S. (2023). Epidemiology of Eating Disorders: Population, Prevalence, Disease Burden and Quality of Life Informing Public Policy in Australia—A Rapid Review. J. Eat. Disord..

[86] Mihaileanu F.V., Fadgyas Stanculete M., Gherman C., Brata V.D., Padureanu A.M., Dita M.O., Turtoi D.C., Bottalico P., Incze V., Stancu B. (2025). Beyond the Physical: Weight Stigma and the Bariatric Patient Journey. J. Clin. Med..

[87] Chukwuma O.V., Ezeani E.I., Fatoye E.O., Benjamin J., Okobi O.E., Nwume C.G., Egberuare E.N. (2024). A Systematic Review of the Effect of Stigmatization on Psychiatric Illness Outcomes. Cureus.

[88] Yearwood L., Masood W. (2024). Behavioral Approaches to Obesity Treatment.

[89] Lau D.C.W., Douketis J.D., Morrison K.M., Hramiak I.M., Sharma A.M., Ur E. (2017). 2006 Canadian clinical practice guidelines on the management and prevention of obesity in adults and children. CMAJ.

[90] Puhl R.M., Heuer C.A. (2009). The stigma of obesity: A review and update. Obesity.

[91] Himmelstein M.S., Puhl R.M., Quinn D.M. (2018). Weight Stigma and Health: The Mediating Role of Coping Responses. Health Psychol..

[92] Dandgey S., Patten E. (2023). Psychological Considerations for the Holistic Management of Obesity. Clin. Med..

[93] Sanyal A.J., Kaplan L.M., Frias J.P., Brouwers B., Wu Q., Thomas M.K., Harris C., Schloot N.C., Du Y., Mather K.J. (2024). Triple Hormone Receptor Agonist Retatrutide for Metabolic Dysfunction-Associated Steatotic Liver Disease: A Randomized Phase 2a Trial. Nat. Med..

